# Post-traumatic diaphragmatic herniation of the liver, examined by positron emission tomography: case report

**DOI:** 10.1186/1749-7922-6-30

**Published:** 2011-08-20

**Authors:** Katsutoshi Sato, Kazumasa Orihashi, Yoshiharu Hamanaka, Norimasa Mitsui, Shinji Hirai, Naru Chatani, Takashi Nishisaka

**Affiliations:** 1 Department of Thoracic Surgery, Hiroshima Prefectural Hospital, Hiroshima, Japan, Ujinakanda 1-5-54, Minami-ku, Hiroshima, 734-8530, Japan; 2 Department of Pathology, Hiroshima Prefectural Hospital, Hiroshima, Japan, Ujinakanda 1-5-54, Minami-ku, Hiroshima, 734-8530, Japan; 3 Department of Cardiovascular Surgery, Hiroshima University Hospital, Hiroshima, Japan, Kasumi 1-2-3, Minami-ku, Hiroshima, 734-8551, Japan

**Keywords:** Diaphragmatic herniation, Positron emission tomography: PET, Blunt trauma

## Abstract

We present a case of post-traumatic diaphragmatic herniation of the liver, which mimicked an intrathoracic tumor. After an automobile accident, the patient underwent thoracotomies for hemothorax and lung cancer in the right chest. Seven months later, computed tomography (CT) demonstrated a round tumor in the thorax adjacent to the right diaphragm with a higher density than the liver parenchyma. An intrathoracic tumor including a primary or metastatic lung cancer was suspected. However, positron emission tomography (PET) showed that the uptake of fluorine-18-fluorodeoxyglucose (FDG) was identical to that in the liver, and the tumor appeared to be contiguous with the liver. Thus, we suspected liver herniation. Core needle biopsy revealed liver cells without neoplastic tissue. Upon surgical exploration, herniation of the liver was found and repaired. PET was helpful in providing morphological and functional information leading to accurate diagnosis of liver herniation in this unusual case.

## Introduction

Diaphragmatic herniation of the liver following blunt trauma may develop long after the initial trauma and remain clinically silent. Unless a large portion of liver and/or other abdominal organs are herniated, it is often difficult to distinguish diaphragmatic herniation of the liver from an intrathoracic tumor [1]. Positron emission tomography (PET) imaging using fluorodeoxyglucose (FDG) labeled with the positron-emitter fluorine-18 provides useful information allowing differentiation of benign lesions from malignant ones. However, FDG is a nonspecific marker of malignancy, and uptake may be seen at sites of active inflammation [2], and also from normal metabolically active tissues, such as the liver [3,4]. We report a case of small diaphragmatic herniation of the liver with diagnostic PET and histological findings. We believe this is the first reported case in the literature of PET findings of herniated liver.

### Case

A 68-year-old woman was involved in an automobile accident and was transferred to the emergency department at the Hiroshima Prefectural Hospital. Computed tomography (CT) on admission demonstrated traumatic aortic injury, multiple rib fractures, and bilateral hemo-pneumothoraces as well as a spiculated mass, 2 cm diameter with pleural indentation in segment 6 of the right lung. She underwent emergent repair of the descending aorta and right pleural drainage. On the fourth post-operative day, bloody drainage from the right chest suddenly increased in volume. The patient was taken back to the operating room and at right thoracotomy, a bleeding point was found on the surface of the diaphragm. Hemostasis was established by using polypropylene suture.

Four months later, the size of lung mass was unchanged, and PET showed little FDG uptake. Because malignancy was suspected and her general condition improved, she underwent surgical resection of the tumor. After meticulous dissection, the right lower lobe was partially resected, but systematic lobectomy and radical lymph node dissection was not feasible due to significant adhesion. Histological examination revealed a well-differentiated adenocarcinoma with clear tissue margins.

**Figure 1:**
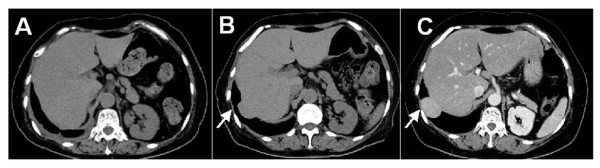
**CT findings of the tumor**. The mass in the right lung field with its inferior border abutting the diaphragm (arrow) increased in size over time. **A **At the first admission. **B **At 3 months, and **C **12 months after the operation for lung cancer.

**Figure 2:**
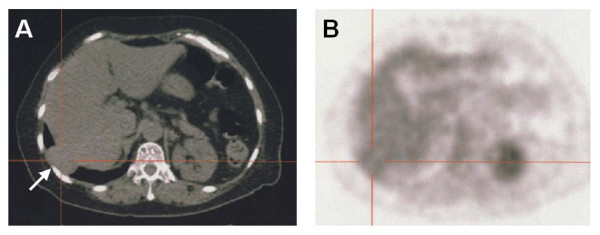
**CT and corresponding PET findings of the tumor**. **A **CT before the operation for liver herniation showed a 3.7 × 2.7 cm solid tumor (arrow). **B **The corresponding PET scan revealed the FDG uptake (SUV max was 3.1), which was equal to the level in liver parenchyma, and contiguous with the liver.

**Figure 3:**
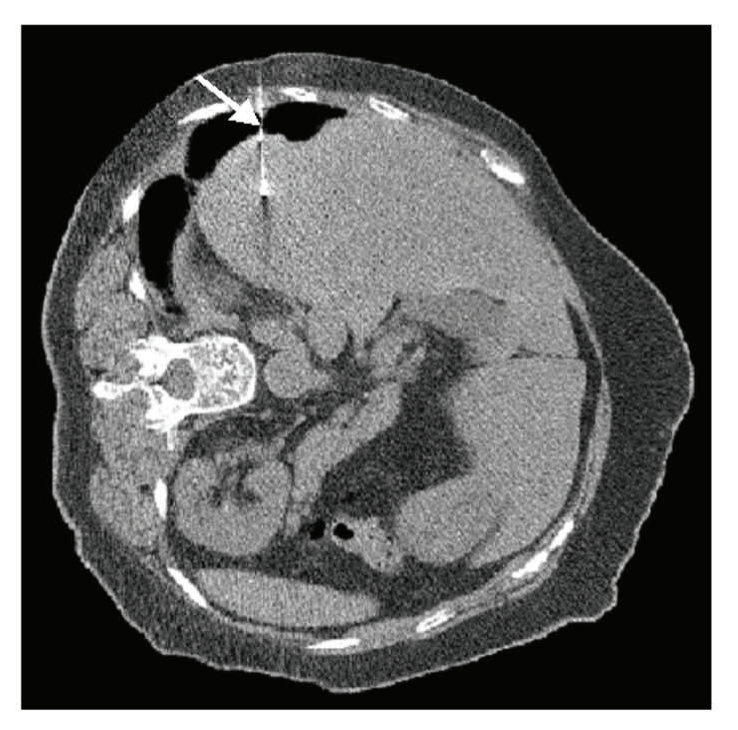
**Percutaneous needle biopsy of the mass**. The biopsy needle penetrated the mass (arrow).

**Figure 4:**
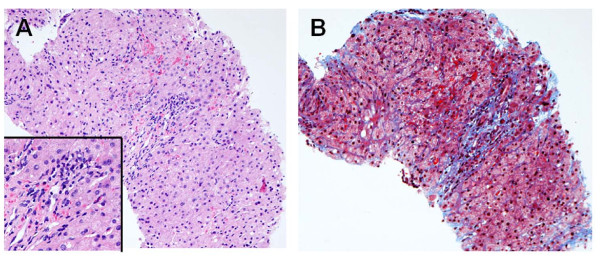
**Histological findings of the tumor**. Histological examination revealed inflammatory cell infiltration around normal liver cells and fibrosis of Glisson's sheath (H & E: **A **×50; inset, ×100. Masson-Trichrome stain: **B **×50).

**Figure 5:**
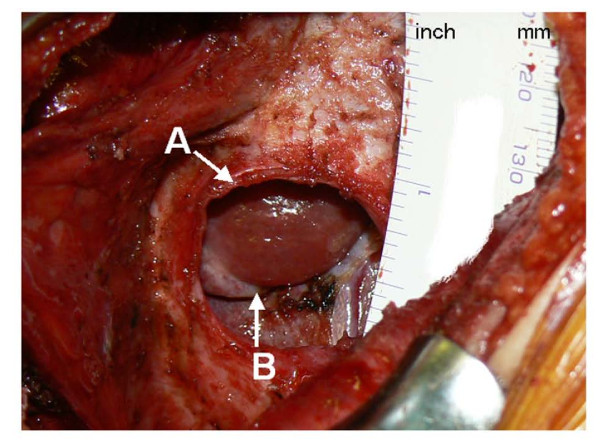
**Intraoperative findings of the herniated liver**. **A **A defect in the right diaphragm. **B **The herniated portion of the liver. The herniated liver surface was congested, compared with surrounding normal liver surface.

The follow-up CT at 3 months revealed another tumor in the right lower lobe adjacent to the diaphragm, which had not been recognized before. Twelve months after lung resection, a discrete ovoid mass 3.7 × 2.7 cm in diameter with slightly higher density than that of liver parenchyma was apparent (Figure 1). Subsequent PET showed FDG uptake in the lesion [the maximum standard uptake value (SUV max) was 3.1] (Figure 2). Metastasis of lung cancer or another heterogenic tumor was entertained as a diagnosis; however, the mass appeared to be contiguous with the liver, which had an identical FDG uptake level. Since liver herniation was suspected, percutaneous needle core biopsy of the mass was performed (Figure 3). The tissue contained only liver cells with inflammatory cell infiltration, and was diagnosed as liver herniation (Figure 4). Because the size of the mass had steadily increased, we elected to perform surgical repair. At operation, diaphragmatic herniation of the liver (3 cm in diameter) was found. The herniated portion of the liver appeared to be congested. As a polypropylene suture was found at the edge of the hernia hilus, we concluded that the hernia had originated from the motor vehicle trauma (Figure 5). The defect was repaired with interrupted sutures. The patient was discharged home after an uneventful recovery and has no evidence of recurrence after two years of follow-up.

## Discussion

Traumatic rupture of the right diaphragm following blunt trauma is uncommon. The extent of herniation varies, from a small portion of liver, to the entire liver plus other abdominal organs. Small herniations are typically asymptomatic, and diagnosis can be delayed for many years [5-7]. The diagnosis can be made when a defect of the diaphragm and/or liver parenchyma is observed on imaging studies such as ultrasonography (US) [8], CT [9], isotopic liver tomogram [10] or magnetic resonance imaging (MRI) [11]. Herniation may be difficult to differentiate from an intrathoracic tumor, especially when only a small portion of the liver is herniated.

In our case, several factors contributed to the difficulty in making an accurate diagnosis of diaphragmatic hernia. These include small herniation of the liver, concomitant lung cancer with suboptimal resection, and elevated CT density in the herniated portion of the liver. At first, as an intrathoracic tumor or metastasis from a lung cancer was suspected, a PET study was performed. Identical FDG uptake in the intrathoracic lesion to that in the liver was seen, leading to a diagnosis of liver herniation. However, since the patient's previous lung cancer showed little FDG uptake, and other neoplasms could not be differentiated solely by PET findings, additional supportive evidence was needed to make a definite diagnosis. US and MRI could not be performed, because of difficulties with the patient's control of breathing during the examination. As the tumor was adherent to the chest wall, we decided to perform a needle biopsy. This provided a conclusive finding of liver cells without neoplastic tissue thus confirming the diagnosis of liver herniation. The CT findings could be explained by strangulation of the herniated liver likely inducing congestion, which was confirmed at operation. This might have led to the higher density in the herniated portion on CT.

Increased FDG uptake in PET is an important finding for differentiating benign lesions from malignant ones and is interpreted by calculation of the SUV [12]. However, FDG is a nonspecific marker of malignancy, and FDG uptake can be seen in normal metabolically-active tissue such as the liver [3,4], or may be caused by active inflammation, probably due to uptake by activated macrophages and inflammatory cells [13].

In this case, histological examination of the specimen by needle biopsy revealed inflammatory cell infiltration around normal liver cells and fibrosis of Glisson's sheath. Yoshimura et al. [14] reported a case in which a herniated liver was resected with histological findings similar to those in our case, without a history of viral and/or other hepatitis. This inflammatory response was likely caused by repeated and sustained mechanical stress upon the herniated portion of the liver. However, it did not show increased FDG uptake above the normal liver level on PET. It is likely that the inflammation might not have been severe enough to induce increased FDG uptake.

Since this report involves only one patient, and there are no other reports in the literature, we cannot assume that herniated liver always exhibits FDG uptake at the same level as liver parenchyma. Hepatic hernias should be included in the differential diagnosis of a right basal mass in the thorax, in the patient with a history of thoraco-abdominal trauma. Recently, PET study has been used frequently in the differential diagnosis of intrathoracic neoplasms. The authors believe that knowledge of this case will be important for diagnosis and decision-making in other cases of ambiguous intrathoracic masses.

## Conclusion

We present a case of post-traumatic diaphragmatic herniation of the liver masquerading as an intrathoracic mass. Although the herniated liver had inflammatory cell infiltration, PET did not show increased FDG uptake above that of the normal liver level. In this case, PET information was helpful for diagnosing even a small liver herniation, due to its normal FDG uptake pattern, informing the subsequent management and repair of the diaphragmatic defect.

### Consent

Written informed consent was obtained from the patient for publication of this Case report and any accompanying images. A copy of the written consent is available for review by the Editor-in-Chief of this journal.

## Competing interests

The authors declare that they have no competing interests.

## Authors' contributions

KS, NM, SH, NC and YH participated in the care of the patient, including the operative part.

TN participated in the pathology.

KS wrote the first draft of the manuscript.

KO and YH critically reviewed the manuscript.

All authors read and approved the final manuscript.
